# PDE5 Inhibition Improves Object Memory in Standard Housed Rats but Not in Rats Housed in an Enriched Environment: Implications for Memory Models?

**DOI:** 10.1371/journal.pone.0111692

**Published:** 2014-11-05

**Authors:** Sven Akkerman, Jos Prickaerts, Ann K. Bruder, Kevin H. M. Wolfs, Jochen De Vry, Tim Vanmierlo, Arjan Blokland

**Affiliations:** 1 Department of Psychiatry and Neuropsychology, Faculty of Health, Medicine and Life Sciences, European School of Neuroscience (EURON), Maastricht University, Maastricht, The Netherlands; 2 Department of Neuropsychology and Psychopharmacology, Faculty of Psychology and Neuroscience, European School of Neuroscience (EURON), Maastricht University, Maastricht, The Netherlands; 3 Department of Immunology and Biochemistry, Biomedical research institute, Hasselt University, Hasselt, Belgium; 4 Institute of Clinical Chemistry and Clinical Pharmacology, University Hospital Bonn, University of Bonn, Bonn, Germany; Sapienza University of Rome, Italy

## Abstract

Drug effects are usually evaluated in animals housed under maximally standardized conditions. However, it is assumed that an enriched environment (EE) more closely resembles human conditions as compared to maximally standardized laboratory conditions. In the present study, we examined the acute cognition enhancing effects of vardenafil, a PDE5 inhibitor, which stimulates protein kinase G/CREB signaling in cells, in three different groups of male Wistar rats tested in an object recognition task (ORT). Rats were either housed solitarily (SOL) or socially (SOC) under standard conditions, or socially in an EE. Although EE animals remembered object information longer in the vehicle condition, vardenafil only improved object memory in SOL and SOC animals. While EE animals had a heavier dorsal hippocampus, we found no differences between experimental groups in total cell numbers in the dentate gyrus, CA2–3 or CA1. Neither were there any differences in markers for pre- and postsynaptic density. No changes in PDE5 mRNA- and protein expression levels were observed. Basal pCREB levels were increased in EE rats only, whereas β-catenin was not affected, suggesting specific activation of the MAP kinase signaling pathway and not the AKT pathway. A possible explanation for the inefficacy of vardenafil could be that CREB signaling is already optimally stimulated in the hippocampus of EE rats. Since previous data has shown that acute PDE5 inhibition does not improve memory performance in humans, the use of EE animals could be considered as a more valid model for testing cognition enhancing drugs.

## Introduction

Many pharmacological animal studies have shown the beneficial effects that drugs can have on memory performance. However, the effects of these drugs are generally only modest, or even absent, when tested in humans. Many factors could explain this possible discrepancy in the effectiveness of drugs on memory performance [1]. It could be argued that the test paradigms used in animal research have poor translational value. Additionally, laboratory rodents may not be suitable for testing cognition enhancing drugs because the impoverished environment in which the animals are raised does not enable the kind of natural brain development seen in humans. The testing of drugs that improve brain function should ideally be done in subjects that have a ‘normal’ brain development [2]–[5]. One way to achieve this is by testing drugs in animals raised in an enriched environment (EE), which has been shown to markedly reduce abnormal repetitive behaviors without affecting the precision or reproducibility of results [6].

From the early studies of Rosenzweig, it is known that environmental enrichment in rats has clear effects on brain development. The ‘EE’ brain is heavier and has more neuronal connections compared to brains of standard housed animals [7]. Related to this, several morphological changes, associated with memory performance, have been observed after EE. For example, EE has been shown to increase; neurogenesis in the dendate gyrus [8], [9], neuronal cell volume, dedritic length/branching/spine density in the CA1 pyramidal neurons [10], [11] and pre- and post-synaptic proteins in the forebrain, hippocampus, thalamus and hypothalamus [12]. Not surprisingly, it is well documented that enriched animals generally outperform animals that are raised under ‘standard’ housing conditions on a wide variety of cognitive behavioral tests [13]–[18]. EE has been shown to affect multiple neurotransmitter systems, including the dopaminergic [19], serotonergic [20], glutamatergic [21], and the cholinergic system [22], which are heavily involved in learning and memory processes [23]. It seems plausible to assume that drugs targeting these neurotransmitters, or their downstream targets, may have differential effects in animals that are living in different housing conditions. This assumption has been confirmed by studies showing that drugs targeting the dopaminergic [24], [25], serotonergic [26], glutamatergic [27] systems, indeed, have differential effects in animals raised in an EE compared to standard housed animals. Although several studies have investigated EE-drug interactions in various neurodegenerative and psychiatric animal models [28], the effects of EE on cognition enhancing drugs have not yet been thoroughly investigated.

In the present study, we examined the efficacy of vardenafil in EE rats and rats that were kept either solitary (SOL) or socially (SOC) in a standard environment. First, we used the object recognition task (ORT) to investigate the effects of the 3 different housing conditions on memory performance, cf. Simpson and Kelly [29]. Subsequently, we tested the acute effect of different doses of the cognition enhancer vardenafil on retention intervals of 24 h and 48 h. Based on previous studies, we hypothesized that EE would enhance ORT performance [5], [9], [30]–[33] and alter the efficacy of vardenafil treatment. After the behavioral testing, brain plasticity was assessed with morphological and neurochemical signaling markers.

## Methods

### Animals

**Figure 1:**
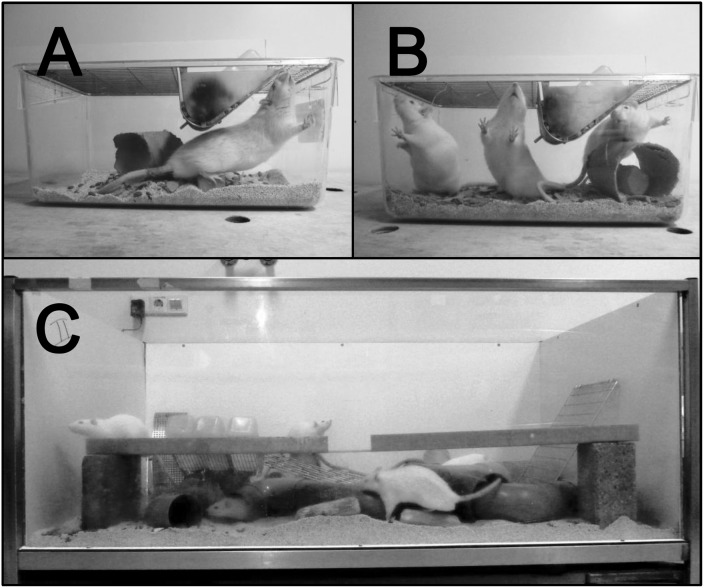
Housing conditions. Pictures of the 3 different housing conditions; solitary housed animals (A), socially housed animals (B), and the animals housed in an enriched environment (C). Of note, the enriched environment picture only represents the object arrangement during one particular week, the constellation and number of items was changed weekly.

This study was approved by the local animal experimental committee of Maastricht University, the Dier Experimenten Commissie (DEC) Maastricht. Fifty-eight male Wistar rats were obtained from Harlan (Horst, The Netherlands) just after weaning (four weeks of age). Eighteen were housed in an enriched environment (EE, n = 9/cage), 18 were socially housed (SOC, n = 3/cage) in standard polycarbonate cages (Tecniplast, type 2154F, dimensions 48×26.5×21 cm, floor area 940 cm^2^), and 22 were individually housed in standard cages (SOL). The 3 different housing conditions are presented in Figure 1. The standard cages contained a cardboard tube, a wooden block, and had a stainless steel wire lid (Tecniplast, series -114) on top. The special EE cages measured 150×90×80 cm and contained a wide array of objects which were rearranged weekly. All cages contained sawdust bedding and animals had free access to food and water. Animals were housed and tested in the same room, under a reversed light/dark cycle (lights on from 19∶00 to 7∶00). A radio always played softly in the background, room temperature was 21°C and the average humidity was kept between 45–55%. Testing was done during the dark phase, between 8∶30 and 16.30. Of note, during testing EE animals were briefly (for about 1 h) moved into a standard holding cage (n = 3/cage).

### Object Recognition Task

Behavioral testing started when the animals were 3 months old. An ORT session consisted of a learning- (T1) and a test-trial (T2), separated by a retention interval. In T1, two identical objects were placed in the arena and in T2 the arena contained a novel (different) object and a copy from T1. In both trials, the animals were allowed to freely explore the objects and the arena for 3 min, during which the exploration of each object was scored manually on a personal computer. The relative discrimination index (d2) was used to assess memory performance. The d2 measure is calculated from the exploration of the objects in the second trial ((time novel object–time familiar object)/(time novel object+time familiar object)). For an elaborate description of the ORT setup, objects and procedures, see Akkerman et al. [34].

Before drug testing started, we examined the performance of the different groups at a 1 h and a 24 h retention interval, which typically relates to a good and poor performance, respectively [35]. During the experiment, a decision was made to additionally test the groups at a 48 h retention interval, as vehicle treated EE rats still showed object memory at the 24 h retention interval.

### Drug Treatment

Vardenafil (kindly donated by Bayer AG, Wuppertal, Germany) was freshly suspended in 1% tylose (methyl-cellulose) in milli-Q, on each experimental day. Vardenafil was tested at different doses: 0 (vehicle), 0.03, 0.1, 0.3 and 1 mg/kg. Administrations were always p.o. (injection volume 2 ml/kg) immediately after the first trial.

### Dorsal Hippocampal Weight

Animals were sacrificed 2 weeks after the behavioral testing. Half of the animals in each housing group were killed by decapitation and the dorsal hippocampus was dissected (on ice), weighed, and immediately frozen in liquid nitrogen. Samples were then stored at −80°C and later used for qPCR and Western Blotting. We focused on the dorsal hippocampus as it is preferentially involved in memory processes, in contrast to the ventral hippocampus which is preferentially involved in emotional processes [36]. Furthermore, there are strong indications for involvement of the dorsal hippocampus in our version of the ORT [37].

### Perfusions

The other half of the animals was killed by intracardial perfusion under lethal pentobarbital anesthesia (100 mg/kg, i.p.). Ice cooled tyrode solution was used during the first minute of perfusion, followed by a fixative containing 4% paraformaldehyde, 15% picric acid, and 0.05% glutaraldehyde in phosphate buffer (0.1 M) [38], for 10 min. Brains were dissected and post-fixated for 18 hours in Somogyi’s fixation solution without the glutaraldehyde. For cryoprotection, the brains were immersed in a 10% sucrose solution in 0.1 M phoshate-buffered elix for 24 h, followed by 24 h immersion in a 20% sucrose solution in 0.1 M phoshate-buffer. Subsequently, the brains were frozen using CO_2_ gas and cut into coronal sections (10 series, thickness 30 µm) at −25°C using a CM3050 cryostat (Leica, Wetzlar, Germany). Sections were stored at −80°C until further processing for stereological measurements.

### Histology and Stereology

Nissl staining was performed on one series of the coronal sections. Sections were put in a 1x TBS solution and mounted on coated glass slides. After mounting, the glass slides were left to air-dry overnight at room temperature. Sections were immersed in a 1% acetic acid, 0.04 M sodium acetate solution (solution A) for 20 min, followed by immersion in a 75% ethanol, 1.25% triton x-100 (Solution B) solution for 20 min, after which the sections were immersed in solution A again, for 20 min. Subsequently, sections were stained by immersion into solution A, containing 0.1% cresyl violet, for 10 min. After staining, sections were washed three times by immersion into solution A for 1 min and dehydrated via immersion into 100% ethanol for 1 min, thrice. Next, the sections were immersed into isopropanol, 2×5 min, followed by immersion in xylene, 2×5 min. Finally, Depex was used to close the sections and fix the cover glasses.

The sections were investigated using design-based stereology [39], a well established method for achieving reliable and accurate estimates of the volume of brain regions and the amount of neurons within it. For volume determination and cell counts of the dorsal hippocampal subregions, the Nissl stained sections ranging from bregma −2.52 to −4.68 mm [40] were analyzed under an Olympus AX70 bright field microscope (analySIS; Imaging System, Münster, Germany). On average, six sections (at 300 µm intervals) were analyzed per animal, depending on the individual rostrocaudal extension of the dorsal hippocampus. Several sub-regions within the dorsal hippocampus were assessed; the dentate gyrus (DG), cornu ammonis 1 (CA1) and cornu ammonis 2 and 3 together (CA2–3). Stereo Investigator software (Version 8.26, MBF Bioscience, Williston, VT, USA) was used to measure the surface area, section thickness, and estimate the cell population of each sub-region. Delineations of each sub-region were made using a 4x objective (numerical aperture [NA] = 0.16) and cell counts were performed using a 100x oil lens (NA = 1.35, Olympus UPlanApo).

### Quantitative PCR

RNA in the dorsal hippocampus was isolated from half the animals of each housing group. Quantitative PCR (qPCR) of *Pde5* was performed to compare the levels of PDE5 mRNA between the different housing conditions. RNA isolation was performed by TRIzol reagent mediated extraction, according to the manufacturer’s instructions (Life Technologies, Bleiswijk, The Netherlands). 500 ng of total RNA was converted into cDNA using iScript Advanced cDNA Synthesis Kit for RT-qPCR (Bio-Rad Laboratories, Veenendaal, The Netherlands). Quantitative PCR of *Pde5* and the housekeeping gene *Hprt* were performed with the IQ SYBR Green Supermix Kit (Bio-Rad Laboratories, Veenendaal, The Netherlands), 0.2 µl primer (10 µM) was added to 4.6 µl H_2_O and 10 µl SYBR Green per 5 µl cDNA sample (2 ng/µl). The following sequences (Sigma-Aldrich, St. Louis, MI) were used: *Hprt*, 5′TTGCTGGTGAAAAGGACCTC3’; *Hprt-1*, 5′ TCCACTTTCGCTGATGACAC 3′; *Pde5*, 5′ TGGTGACGTTAGAGGTCCTG3’; *Pde5*, 5′CGCTGTTTCCAGATCAGACA3’. Analysis of the data was performed via the delta-delta CT method and expression of *Pde5* was normalized against *Hprt*.

### Western Blotting

Western Blots were used to assess protein levels of BDNF, PDE5, pCREB, β-catenin, pGSK-3β, synaptophysin, PSD95, and ERK2 in the dorsal hippocampus of half of the animals of each housing condition. GAPDH and β-actin were used for normalization.

One hundred to 130 mg dorsal hippocampus tissue was homogenized using a Mini-Bead Beater three times for 30 s in 1 ml ice-cold homogenizing buffer (two phosphatase inhibitor tablets/20 ml ((Roche, #04906845001, Almere, The Netherlands) and one protease inhibitor tablet/20 ml (Roche #11836153001, Almere, The Netherlands) were added to the homogenizing buffer (100 mM Tris, 200 mM NaCl, 1 mM EDTA, 2 mM DTT, 0.05% Triton (vol/vol)). Samples were centrifuged for 20 min at 4°C, 16000 g and the supernatant was stored at −80°C. Protein concentrations were determined using Bio-Rad Lowry Protein Assay (Bio-Rad Laboratories Inc., Hercules, USA).

Brain homogenates in sample buffer were boiled for five minutes and then separated on a 10% or 14% (mBDNF and proBDNF) SDS–PAGE gel (30 µg per sample). Following electrophoresis, proteins were transferred to a nitrocellulose membrane (Bio-Rad Laboratories, Hercules, USA), which was subsequently blocked with blocking buffer (50% Odyssey blocking buffer in PBS, Li-Cor, Lincoln, USA) for one hour at room temperature. Next, the membranes were incubated overnight at 4°C with the primary antibodies in blocking buffer: 1∶600 rabbit anti-BDNF (H-117; Santa Cruz Biotechnology #SC-20981, Heidelberg, Germany); 1∶500 rabbit anti-PDE5A1 (Fabgennix #PC-Pde5A, Frisco, TX), 1∶100 rabbit anti-pCREB (Cell Signaling Technology #9198S, Beverly, MA), 1∶2000 mouse anti- β -catenin (BD Transduction laboratories #20079, Franklin Lakes, NJ), 1∶1000 rabbit anti-pGSK-3β (Cell Signaling Technology #9336S, Beverly, MA), 1∶1000 mouse anti-synaptophysin (Millipore MSxsynaptophysin MS#MAB5258, Billerica, MA), 1∶2000 mouse anti-PSD95 (QED Bioscience inc #56452, San Diego, CA), 1∶1000 rabbit anti-ERK2 (Cell signaling technology #9108, Beverly, MA). For normalization, 1∶2.000.000 mouse anti-Gapdh (Fitzgerald Industries #10R-G109A, Huissen, The Netherlands) or 1∶1000 mouse anti-β-actin (Santa Cruz Biotechnologies #F0110; Santa Cruz, CA) was used. After washing with phosphate-buffered saline-0.1%Tween (PBS-T), membranes were incubated for 1 hour at room temperature with the following secondary antibodies in blocking buffer: 1∶5000 goat anti-rabbit IRDye 800 (Li-Cor #926-32211) and 1∶5000 donkey anti-mouse IRDye 680 (Li-Cor #926-32222). Membranes were washed in PBS-T and fluorescent bands were visualized using an Odyssey Infrared Imaging System (Li-Cor). Intensities of specific bands were quantified using ImageJ (http://rsbweb.nih.gov/ij/), corrected for background signal and GAPDH signal.

Within the BDNF blot, different BDNF domains were analyzed, distinguishing between mBDNF (14 kDa) and proBDNF (16 kDa), as previously described [41], [42]. The PDE5 antibody reportedly recognizes the two isoforms of PDE5A1 (99 kDa) and PDE5A2 (89 kDa), yet only the 99 kDa was detectable in our blots.

### Statistical Analysis

The basic readout parameter of the ORT is the time spent exploring each object during a trial. Trials in which animals had explored both objects for less than 6 s in T1 or 9 s in T2 were excluded from analysis, since these data may not be reliable [34]. We examined the effects of retention delay on exploration (e1 and e2) by performing a two-way ANOVA (Interval×Housing) on all the vehicle treated conditions. The effect of the vardenafil treatment on exploration was also investigated using two-way ANOVA (Treatment×Housing), though only in the 24 h interval. In the 48 h interval, the effects of vardenafil treatment were examined using one-way ANOVAs within each separate housing condition, because different doses were tested in each housing condition at the 48 h retention interval. Significant differences were further investigated using Tukey HSD post-hoc tests.

The effects of retention delay on object discrimination performance (d2) were examined using a two way ANOVA (Interval×Housing) on all vehicle treated conditions. Housing effects on d2 were further evaluated for each retention interval separately, using one-way ANOVA followed by Tukey HSD post-hoc tests. Effects of vardenafil on object discrimination were assessed by performing one-way ANOVA’s on each housing condition separately, within the 24 h and 48 h retention intervals. Significant effects were further investigated with Tukey HSD post-hoc tests, comparing each different dose with the vehicle condition. In order to examine whether the animals actually showed object discrimination, the d2 of each individual condition was also compared to 0 using one-sample *t*-tests.

Differences in hippocampal weight, stereological- and biochemical measures were also analyzed using a one-way ANOVA and post-hoc Tukey HSD tests.

## Results

### Object Recognition Task: Effects of Housing on Exploration

The exploration data of the animals is shown in Table 1. A two-way ANOVA (Interval×Housing) was performed, incorporating only vehicle treated groups. Significant main effects were found for both Interval and Housing on e1 (*F’s*(2,161) >10.17, *p’s*<0.001) and e2 (*F’s*(2,161) = 17.94, *p’s*<0.001), as well as significant Interval by Housing interaction effects (*F’s*(4,161) >16.16, *p’s*<0.001). Therefore, one-way ANOVAs were performed on each separate retention interval.

**Table 1:** Exploration levels in the ORT.

Retention interval	Vardenafil Dose (mg/kg)	Housing	n	e1	e2
**1** **h Interval**	0	SOL	21	15.34 (1.51)	19.01 (1.30)
	0	SOC	17	43.48 (3.04)	52.08 (4.49)
	0	EE	18	40.10 (4.91)	46.55 (5.14)
**24** **h Interval**	0	SOL	22	29.88 (1.87)	28.45 (2.01)
	0	SOC	18	29.45 (2.44)	30.65 (2.92)
	0	EE	18	19.05 (1.80)	16.28 (1.42)
	0.1	SOL	22	29.91 (2.27)	27.24 (1.49)
	0.1	SOC	18	30.65 (2.85)	27.43 (2.31)
	0.1	EE	17	23.00 (1.93)	22.11 (2.34)
	0.3	SOL	22	29.23 (2.15)	25.83 (1.59)
	0.3	SOC	18	32.28 (3.38)	31.45 (3.15)
	0.3	EE	18	21.79 (3.29)	24.65 (2.27)
	1	SOL	22	29.96 (2.26)	31.74 (2.36)
	1	SOC	18	30.43 (3.15)	36.26 (3.75)
	1	EE	18	21.56 (2.03)	20.77 (2.32)
**48** **h Interval**	0	SOL	21	24.02 (1.53)	28.66 (1.72)
	0	SOC	18	23.15 (1.06)	33.82 (2.07)
	0	EE	17	23.52 (2.56)	30.38 (3.04)
	0.03	EE	16	20.24 (1.16)	17.20 (1.33)
	0.1	EE	17	19.12 (1.49)	20.13 (1.48)
	0.3	SOC	18	24.69 (2.25)	36.84 (2.01)
	0.3	EE	17	25.51 (3.27)	24.12 (2.76)
	1	SOL	22	30.05 (1.84)	30.92 (1.77)
	1	EE	15	26.17 (2.23)	26.28 2.97)

This table shows the number of animals (n) and mean exploration time (± SEM) in seconds on both the objects in T1 (e1) and T2 (e2) in every treatment condition. SOL, SOC, and EE animals were tested repeatedly, so every individual animal performed each experimental condition once. The 1 h retention interval was tested first, followed by the 24 h interval conditions. Finally, the 48 h retention interval conditions were tested. Of note, although a total number of 22 SOL, 18 SOC and 18 EE animals were tested in each condition, animals that explored less than 6 s in T1 and/or less than 9 s in T2 were excluded from the analyses.

At the 1 h interval, one-way ANOVA revealed significant differences between vehicle treated housing conditions on e1 (*F*(2,53) = 22.59, *p*<0.001) and e2 (*F*(2,53) = 23.36, *p*<0.001). Tukey HSD post-hoc tests showed that SOL animals had a lower e1 and e2 compared to the SOC and EE animals. At the 24 h retention interval one-way ANOVA also revealed significant differences on e1 (*F*(2,55) = 8.72, *p*<0.01) and e2 (*F*(2,55) = 18.85, *p*<0.001) between the vehicle treated animals. Post-hoc analysis demonstrated that exploration levels (e1 and e2) were lower in vehicle treated EE animals as compared to the two other housing conditions. At the 48 h retention interval, no significant differences between the housing conditions were found (*F*’s(2,53) <1.38, n.s.).

### Object Recognition Task: Effects of Vardenafil Treatment on Exploration

A Housing×Treatment ANOVA was performed to investigate the effects vardenafil treatment on exploration levels in the 24 h retention interval. Because differed doses of vardenafil were tested in each housing condition of the 48 h interval, one-way ANOVA was used to separately analyze their effects within each housing condition.

### 24 h Retention Interval

Housing×Treatment ANOVA at the 24 h retention interval showed that there were no overall Treatment effects on e1 or e2 (*F*(3,219) = 0.31 and *F*(3,219) = 2.18, both n.s.). There were, on the other hand, significant overall effects of Housing on exploration (e1: *F*(2,219) = 16.31, e2: *F*(2,219) = 19.39, both *p*<0.001) and Treatment by Housing interaction effects (*F*’s (6,219) <1.71, n.s.). Post-hoc analysis with Tukey HSD tests showed that, on the 24 h retention interval, the overall exploration (both e1 and e2) of EE animals was lower compared to SOL and SOC animals.

### 48 h Retention Interval

In the SOL animals, one-way ANOVA showed that animals treated with 1 mg/kg vardenafil had a higher e1 compared to vehicle treated animals (*F*(1,41) = 6.30, *p*<0.05) and that no differences were present on e2 (*F*(1,41) = 0.84, n.s.). In the EE animals, no effect was found of Treatment on e1 (*F*(4,77) = 1.85, n.s.), however, there was an effect of Treatment on e2 (*F*(4,77) = 4.54, *p*<0.01). Post-hoc analysis showed that EE animals treated with 0.03 and 0.1 mg/kg vardenafil had a lower e2 compared to vehicle treated EE animals. Finally, in SOC animals, no effects of vardenafil treatment on exploration levels were found (*F*’s<1.10, n.s.).

### Object Recognition Task: Effects of Retention Delay on Object Discrimination

**Figure 2:**
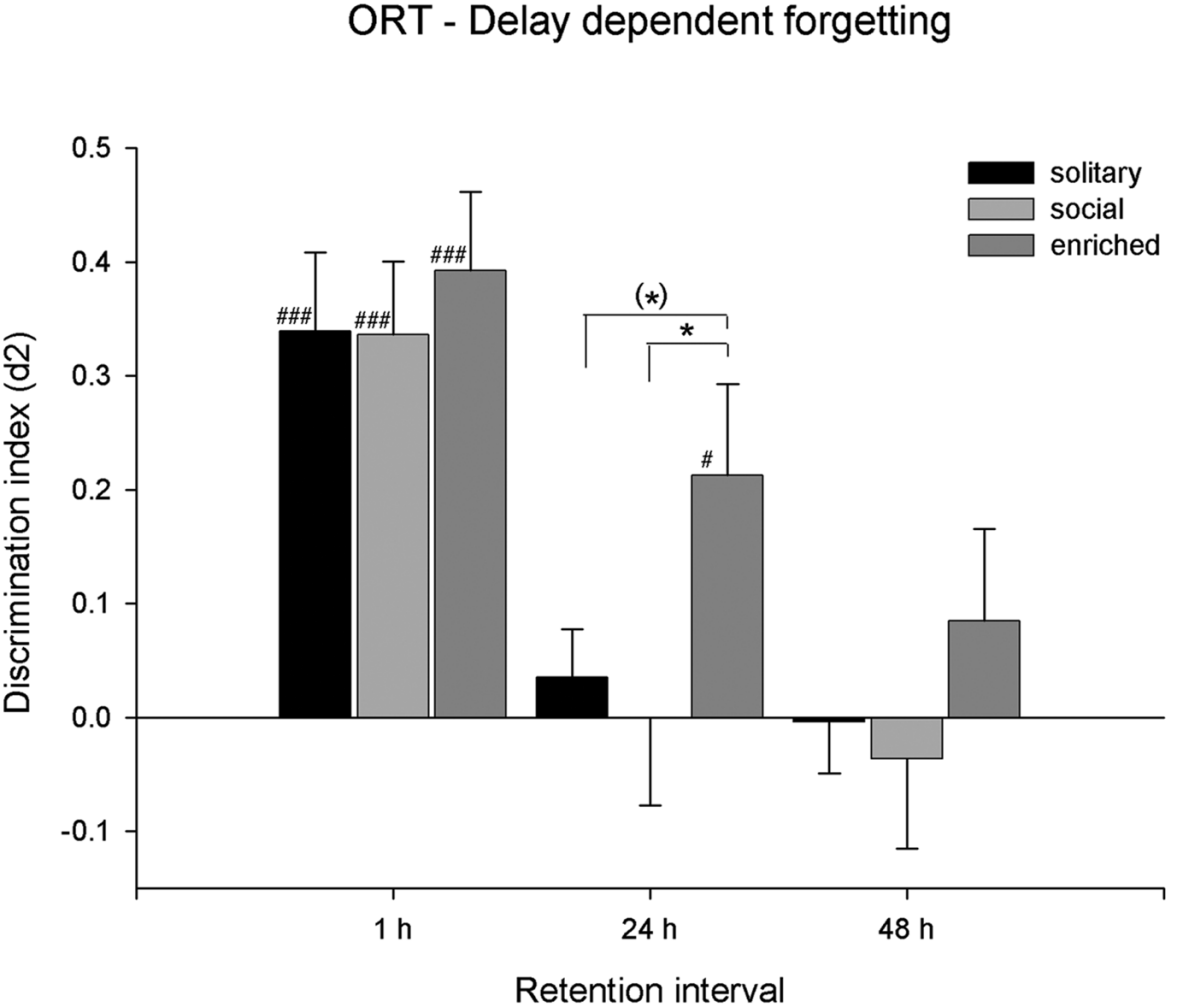
Effect of retention delay on object discrimination. Discrimination performance (d2, mean + SEM) of SOL, SOC and EE rats treated with vehicle in a 1 h, and 24 h and 48 h retention interval (x-axis) in the object recognition task. A difference from zero is indicated with hash symbols (one-sample *t*-test; #: *p*<0.05; ###: *p*<0.001), differences between conditions within the same retention interval are indicated with asterisks (Tukey HSD; (*): p<0.10; *: *p*<0.05).

**Figure 3:**
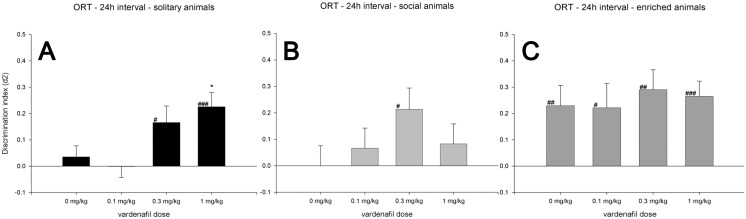
ORT 24 h intervals. Discrimination performance (d2, mean + SEM) of SOL (A), SOC (B) and EE (C) rats after administration of different doses of vardenafil in the object recognition task. All housing conditions were tested in a 24 h retention interval in combination with 0, 0.1, 0.3 and 1 mg/kg vardenafil (x-axis). A difference from zero is indicated with hash symbols (one-sample *t*-test; #: *p*<0.05; ##: *p*<0.01; ###: *p*<0.001), differences from the vehicle condition (0 mg/kg) within the same retention interval are indicated with an asterisk (Tukey HSD; *: *p*<0.05). Of note, the 0 mg/kg vardenafil conditions are identical to those used in Figure 2.

**Figure 4:**
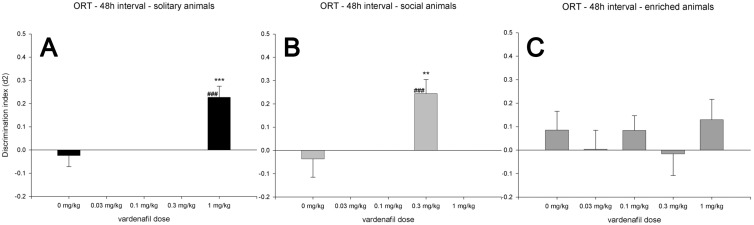
ORT 48 h intervals. Discrimination performance (d2, mean + SEM) of SOL (A), SOC (B) and EE (C) rats after administration of different doses of vardenafil in the object recognition task. All housing conditions were tested in a 48 h retention interval in combination with 0, 0.1, 0.3 and 1 mg/kg vardenafil (x-axis), EE animals also received 0.03 mg/kg vardenafil. A difference from zero is indicated with hash symbols (one-sample *t*-test; ###: *p*<0.001), differences from the vehicle condition (0 mg/kg) within the same retention interval are indicated with asterisks (Tukey HSD; **: *p*<0.01; ***: p<0.001). Of note, the 0 mg/kg vardenafil conditions are identical to those used in Figure 2.

The discrimination performance of the animals is shown in Figures 2–4. A two-way ANOVA (Interval×Housing) was performed, incorporating only vehicle treated groups. There was a significant main effect of Interval on discrimination performance (*F*(2,161) = 21.33, *p*<0.001). Post-hoc analysis revealed that the overall d2 value was higher at the 1 h interval compared to the 24 h and 48 h intervals. There was also a significant main effect of Housing on d2 (*F*(2,161) = 3.05, *p*<0.05). Post-hoc analysis revealed that EE animals showed better overall object discrimination (d2 was higher) compared to SOC animals. No significant Interval by Housing interaction effects were found for d2 (*F*(4,161) = 0.50, n.s).

One-way ANOVA’s were performed on the vehicle conditions of each separate retention interval. No significant d2 differences were found between the housing conditions at the 1 h (*F*(2,53) = 0.19, n.s.) and 48 h (*F*(2,53) <0.63, n.s.) retention intervals. However, there were significant differences between the d2 measures at the 24 h interval (*F*(2,55) = 3.49, *p*<0.05). Post-hoc analysis revealed that the d2 value of EE animals was higher at the 24 h retention interval, compared to SOC animals.

One-sample *t*-tests on the vehicle treated conditions showed that, at the 1 h retention interval, the d2 measure was significantly higher than zero in all vehicle treated housing conditions. Yet, after the 24 h retention interval, only EE animals still discriminated between the novel and familiar objects. After the 48 h retention interval, none of the groups still showed significant object discrimination. The effects of retention delay on the different housing conditions are graphically presented in Figure 2.

### Object Recognition Task: Effects of Vardenafil on Object Discrimination

Because different doses of vardenafil were used in the 24 h and 48 h retention intervals, the effects were analyzed separately, using one-way ANOVAs for each housing condition/retention interval. Of note, the vehicle groups used in this section are the same as those in the section ‘Object recognition task: effects of retention delay’.

### 24 h Retention Interval

In the 24 h interval, one-way ANOVAs within each separate Housing condition revealed no significant differences between d2 values of the different vardenafil treatments (*F*’s<0.34), with the exception of the SOL animals (*F*(3, 84) = 4.35, *p*<0.01). Post-hoc analysis showed that the d2 of SOL animals treated with 1 mg/kg vardenafil was increased compared to vehicle treated animals.

One-sample *t*-tests showed that SOL animals treated with 0.3 or 1 mg/kg vardenafil had a d2 value significantly higher than zero. The same was true for SOC animals treated with 0.3 mg/kg vardenafil. In EE animals, the vehicle condition and all of the included vardenafil doses resulted in a d2 that was higher than zero. The effects of vardenafil in the 24 h retention interval are graphically presented in Figure 3.

### 48 h Retention Interval

In SOL and SOC animals, only the most effective vardenafil dose from the 24 h interval was re-tested in the 48 h retention interval. Multiple vardenafil doses were tested in EE animals. We analyzed the effects of vardenafil treatment within each housing condition using one-way ANOVA. This revealed an effect of Treatment on the discrimination (d2) performance in the SOL animals (*F*(1,41) = 12.13, *p*<0.001) and the SOC animals (*F*(1,34) = 7.92, *p*<0.01). Thus, after 48 h hours, object discrimination was still improved by the vardenafil doses of 1 mg/kg and 0.3 mg/kg in SOL and SOC animals, respectively. However, in EE animals, one-way ANOVA revealed no significant Treatment effect on discrimination performance (d2) (*F*(4,77) = 0.58, n.s.).

One-sample *t*-tests showed that the d2 of the SOL and SOC differed from zero at a dose of 1 mg/kg and 0.3 mg/kg vardenafil, respectively. However, none of the tested vardenafil doses improved the discrimination performance of the EE animals to a level above zero in the 48 h retention interval. The discrimination performances of the housing conditions in the 48 h retention interval are graphically presented in Figure 4.

### Dorsal Hippocampal Weight

One-way ANOVA revealed significant differences in dorsal hippocampal weights between the different housing conditions (*F*(2,26) = 4.36, *p* = 0.023). Post-hoc analysis revealed that EE animals had a heavier dorsal hippocampus, though only compared to SOC animals. However, there was also a strong trend (*p* = 0.052) towards a significant difference between EE and SOL animals (Table 2/Figure S1).

**Table 2:** Results stereological and biochemical measurements.

Housing Condition	Supplementary Material		SOL	SOC	EE
Weight dorsal hippocampus (mg)	Figure S1		69.3 (2.60)^(^*^)^	67.9 (2.60)+	72.0 (3.60)^(^*^)^/+
Volume dorsal hippocampus (mm^3^)	Figure S2A	DG	0.63 (0.02)***/##	0.56 (0.01)##	0.51 (0.01)***
		CA1	0.30 (0.01)	0.32 (0.02)	0.31 (0.02)
		CA2–3	0.59 (0.01)	0.71 (0.03)	0.61 (0.03)
Cells dorsal hippocampus (×10^6^)	Figure S2B	DG	0.53 (0.03)	0.59 (0.02)	0.52 (0.06)
		CA1	0.30 (0.02)	0.38 (0.05)	0.37 (0.04)
		CA2–3	0.39 (0.03)	0.54 (0.09)	0.43 (0.04)
Density dorsal hippocampus (×10^6^/mm^3^)	Figure S2C	DG	0.84 (0.05)	1.06 (0.03)	1.02 (0.13)
		CA1	1.02 (0.08)	1.19 (0.12)	1.16 (0.07)
		CA2–3	0.66 (0.06)	0.74 (0.10)	0.71 (0.05)
PDE5 mRNA (PDE5 mRNA/HGPRT)	Figure S3A		1.00 (0.06)	1.09 (0.04)	1.19 (0.11)
PDE5 protein (PDE5/β-actin)	Figure S3B		0.86 (0.08)	0.80 (0.05)	0.80 (0.08)
Synaptophysin (Synaptophysin/β-actin)	Figure S4A		1.50 (0.03)	1.43 (0.04)	1.43 (0.06)
PSD95 (PSD95/β-actin)	Figure S4B		0.62 (0.30)	0.64 (0.02)	0.71 (0.01)
proBDNF (proBDNF/β-actin)	Figure S5A		0.51 (0.06)	0.44 (0.04)	0.44 (0.05)
mBDNF (mBDNF/β-actin)	Figure S5B		0.89 (0.08)	0.84 (0.07)	0.69 (0.04)
pGSK-3β 9 (pGSK-3β/β-actin)	Figure S6A		1.24 (0.04)	1.29 (0.09)	1.37 (0.12)
β-catenin (β-catenin/β-actin)	Figure S6B		1.00 (0.07)	1.18 (0.10)	1.10 (0.05)
ERK2 (ERK2/GAPDH)	Figure S7A		0.68 (0.03)***	0.73 (0.01)++	0.87 (0.02)***/++
pCREB (pCREB/GAPDH)	Figure S7B		1.62 (0.09)*	2.04 (0.13)	2.11 (0.20)*

Comparisons of the stereological and biochemical measurements between the different housing conditions using one-way ANOVA and Tukey HSD post hoc analysis. Individual cells show the mean (+SEM) of each housing condition. Significant differences between SOL and EE animals are indicated by asterisks (^(^*^)^: P = 0.052; *: p<0.05; ***: p<0.001), significant differences between SOL and SOC animals are indicated by hash signs (##: p<0.01) and significant differences between SOC and EE animals are indicated by plus signs (+: p<0.05; ++: p<0.01).

### Stereology

The DG, CA1 and CA2–3 of the dorsal hippocampus were compared between the different housing conditions (Table 2/Figure S2). One-way ANOVA revealed no differences on total cell numbers (*F’s*(2,12) <1.50, n.s.) and cell density (*F’s*(2,12) <2.13, n.s.). The only significant difference found was on the volume of the DG region (*F*(2,12) = 19,26, p<0.001) which was larger in SOL animals, compared to both other groups.

### PDE5 mRNA and Protein Expression

One-way ANOVA revealed no differences in PDE5 mRNA expression between the housing conditions in the dorsal hippocampus (*F*(2,24) = 1.41, n.s.; Table 2/Figure S3A). In line with mRNA expression levels, there were also no differences detected between PDE5A1 protein levels between the different housing conditions (*F*(2, 14) = 0.21, n.s.; Table 2/Figure S3B).

### Western Blotting

Differences between the different housing conditions in dorsal hippocampal protein levels were investigated using Western Blots (Table 2). One-way ANOVA between the housing conditions revealed no differences in the levels of synaptophysin (*F*(2,22) = 0.74, n.s.; Figure S4A), PSD95 (*F*(2,26) = 1.99, n.s.; Figure S4B), proBDNF (*F* (2,25) = 0.72, n.s.; Figure S5A), mature BDNF (*F*(2,24) = 1.78, n.s.; Figure S5B), pGSK-3β (*F*(2,22) = 0.46, n.s.; Figure S6A) and β-catenin (*F*(2,24) = 1.32, n.s.; Figure S6B). On the other hand, there were significant differences in the levels of ERK2 (*F*(2,24) = 17.62, *p*<0.001; Figure S7A) and pCREB (*F*(2,26) = 3.79, p<0.05; Figure S7B). Post-hoc analysis showed that ERK2 was increased in EE animals compared to SOL and SOC animals and the expression of pCREB was increased in EE animals, compared to SOL animals.

## Discussion

By analyzing the vehicle conditions, we examined how the different housing conditions affected baseline memory performance of rats in an ORT. One hour after learning, object memory appeared to be equal for all housing groups, as all groups discriminated equally well between the novel and familiar objects. However, after 24 h EE animals outperformed both other groups, which no longer showed any sign of object memory after this retention interval. These findings are in accordance with previous studies [9], [15], [29], [31], [43], demonstrating that EE enhances cognitive performance. After 48 h, none of the housing conditions discriminated between objects anymore, thereby indicating that also the EE animals had also forgotten the object information at this retention interval.

Multiple studies have produced evidence which suggests that vardenafil has memory enhancing properties in SOL and SOC rats [44]–[51]. The main goal of the current study was to investigate whether these memory enhancing effects could be extended to EE rats, as it is known that EE promotes behavior that is more similar to the animal’s natural behavior [52], [53] and may therefore more closely resemble human living conditions [4]. As in previous studies [47]–[50], object memory of SOL animals was effectively enhanced by 1 mg/kg vardenafil in a 24 h retention interval. SOL animals also showed intermediate memory improvement at a dose of 0.3 mg/kg, as performance was above chance level but not improved compared to vehicle-treatment [35]. In SOC animals, an intermediate vardenafil effect was also found with a 0.3 mg/kg dose of vardenafil, yet 1 mg/kg was ineffective. Vehicle treated EE animals still showed object memory after 24 h, though none of the tested vardenafil doses was able to improve memory performance at this retention interval. Thus, vardenafil was ineffective in improving cognition in EE animals, asserted an intermediate effect in SOC animals, and successfully improved memory in SOL animals. Furthermore, the most effective dose in SOC animals was lower and the effective dose-range was smaller compared to SOC animals, demonstrating that the stress of social isolation alone is enough to cause marked differences in the efficacy of vardenafil. The lack of effect in EE animals might be due to a ceiling effect, since the higher performance in the vehicle condition might leave less room for improvement by vardenafil. Hence, it was decided to extend the retention interval to 48 h.

After the 48 h retention interval, there was no more object discrimination in any of the vehicle treated housing conditions. Even after 48 h, memory performance in SOL (1 mg/kg) and SOC (0.3 mg/kg) animals was still fully enhanced by vardenafil. However, like in the 24 h retention interval, none of the tested vardenafil doses caused memory improvement in EE rats, ruling out the possibility of a ceiling effect. The lowered effective dose of vardenafil in SOC animals compared to SOL animals suggests that the effective dose in EE animals might be even lower and the lowest tested dose of 0.03 mg/kg may still have been excessive. On the other hand, considering the low brain-plasma ratio and IC50 of vardenafil, it is questionable as to whether there would still be enough vardenafil available in the brain to be biologically effective with a further lowering of the dose [46]. Alternatively, it could be argued that the effective dose range in EE animals was so narrow that the effect was simply missed. However, based on our previous data with PDE inhibitors in SOL animals, we assume that EE animals are insensitive to vardenafil treatment. This lack of effect of vardenafil on memory in EE rats resembles the ineffectiveness of this drug on human memory performance [54]–[56] and suggests that, compared to SOL/SOC animals, the EE rat could be considered as a more valid animal model for testing cognition enhancing drugs [3], [4].

When animals display a statistically significant preference for the novel objects, it can be safely inferred that they were able to remember/recognize the sample objects. On the other hand, if animals fail to display a preference for the novel object, this may not necessarily indicate an absence of object memory as perceptual, attentional and motivational mechanisms also play an important role in the manifestation of a preference for the novel object [57]–[59]. However, since all housing conditions showed a preference for the novel objects at the 1 h retention interval, we assume that these processes were intact in the current study. In a minority of test conditions, a housing condition or vardenafil treatment appeared to affect exploration levels. However, as previousy reported in other studies [34], [57], exploration levels were not related to object discrimination performance. PDE5-Is have shown performance enhancing effects across a wide variety of memory tasks, including; the Elevated Plus Maze (EPM) [60], Radial Arm Maze (RAM), Morris Water Maze (MWM) [61], [62]. Y-maze [63], [64], Social Transmission Food Preference (STFP) task as well as active- [63] and inhibitory avoidance tests [60], [65]. Therefore, we believe that the observed effects of vardenafil on discrimination performance are a reflection of enhanced memory processes.

The insensitivity of EE animals to vardenafil treatment may be related to changes in brain plasticity, influencing their memory performance. To assess brain plasticity, we analyzed several morphological and neurochemical signaling markers. Based on the enhanced ORT performance and the increased dorsal hippocampal weight in the EE rats, we argue that an effective EE procedure was used in the present study. However, we did not find any effect of Housing in the dorsal hippocampus on absolute cell numbers nor on the synaptic markers synaptophysin and PSD95. Neither did we observe a change in pro- or mature BDNF levels, a neurotrophic protein typically implicated in plastic changes. This appears to be in contrast with the findings of most studies on EE [15], [66]–[70]. However, there are also EE studies that have reported cognitive improvements without any changes in hippocampal BDNF levels [5], [53], [71]–[74], implicating that hippocampal BDNF is not essential for the observed cognition improvement found in EE animals. Of note, discrepancies between EE studies in morphological and biochemical effects can be caused by a variety of factors, such as; exercise level, strain, age, strain, EE protocols and differences in species of the animals involved in the studies [75].

We also assessed two BDNF-related signaling pathways, the MAP kinase/CREB signaling pathway and the AKT/β-catenin pathway. ERK is a kinase, activated by BDNF signaling, which phosphorylates the transcription factor CREB. CREB is involved in the expression of genes important for plasticity (e.g. neurogenesis or synaptogenesis) as well as memory processes [76]. AKT is another kinase activated by BDNF signaling. Dephosphorylated GSK-3β is phosphorylated by AKT and inhibits several proteins [77] like β-catenin which, in its dephosphorylated state, translocates to the nucleus where it induces the transcription of specific genes involved in neurogenesis and synaptogenesis [78], [79]. Thus, phosphorylation of GSK-3β leads to disinhibition of β-catenin, resulting in more neurogenesis or synaptogenesis. AKT/β-catenin signaling appeared unaffected by EE as pGSK-3 β and β-catenin levels were unchanged. Interestingly, MAP kinase/CREB signaling was increased as ERK expression and phosphorylation of CREB were enhanced in the dorsal hippocampus of EE rats. Yet, these increases are apparently not BDNF dependent.

The additional exercise in EE has been shown to be the major stimulant of BDNF increases and neurogenic effects [31], [80], [81], which are assumed to enhance memory performance [82]. However, it has recently been shown that EE in the absence of exercise can also improve object recognition memory, perhaps via mechanisms that are independent of BDNF upregulation and neurogenesis in the dentate gyrus of the hippocampus, as the latter two effects were not observed [31]. Consequently, we argue that in the present study, the level of exercise might not have been high enough to increase BDNF as well as cell numbers and probably also the number of synapses in our animals.

The hippocampus has been shown to contain PDE5 mRNA and protein, though at low levels and in different types of cells [46]. PDE5 mRNA and protein expression were assessed in the dorsal hippocampus. However, no differences were found between the different housing conditions on either of these measures, indicating that the effects of EE on the efficacy of vardenafil cannot be explained by a difference in target enzyme availability.

It has been suggested that the cognition-enhancing effects of vardenafil are related to activation of cellular cGMP/protein kinase G (PKG)/CREB pathways implicated in glutamatergic signaling [48], [83]. As described above, CREB activation is already enhanced in the dorsal hippocampus of EE rats. Therefore, a possible explanation could be hypothesized that vardenafil treatment did not further enhance CREB signaling since it was already fully stimulated in the hippocampus of EE rats.

Taken together, our study showed that baseline memory performance in the ORT was better in EE rats compared to SOL and SOC animals, i.e. they were able to remember object information over a longer period of time. However, no effects were found in the dorsal hippocampus on BDNF levels, number of cells and markers for synapses. Furthermore, the effectiveness of vardenafil diminished with the level of enrichment, yet the target of vardenafil, PDE5, was unaffected by EE. Therefore, the effect of EE on vardenafil efficacy is probably due to a more general mechanism, which means that other classes of drugs are likely to be affected as well. If EE reduces the efficacy of drugs via widespread, general molecular memory mechanisms, this might partly explain the discrepancy in success rate between pre-clinical drug studies in animals and studies performed in human subjects. EE may provide a more valid and predictive animal model for pre-clinical drug screening compared to highly impoverished standard housing conditions. Additionally, it would significantly increase animal welfare [6]. For future studies, it would be interesting to investigate whether drugs that improve memory performance in humans are also effective in EE rats. This would provide further insight into the validity of SOL/SOC versus EE rats as an animal model for testing cognition enhancing drugs.

## Supporting Information

Figure S1 **Dorsal hippocampal weights.** Dorsal hippocampal weights of the three experimental groups in grams (mean + SEM). Significant differences between groups are indicated with asterisks (Tukey HSD; (*): *p* = 0.052; *: *p*<0.05).
(TIF)

Figure S2 **Stereology.** The volume (mm^3^) total number of cells and cell density (number of cells/mm^3^) in the DG, CA1 and CA2–3 sub-regions of dorsal hippocampus of solitary- (SOL), social- (SOC) and environmentally enriched (EE) animals. One-way ANOVA only revealed significant differences between the housing conditions in the DG, these are indicated with asterisks (Tukey HSD; **: p<0.01; ***: p<0.001).
(TIF)

Figure S3 **PDE5 mRNA and protein concentration in the dorsal hippocampus.** (A) qPCR analysis showing mRNA levels of PDE5 of the three experimental conditions. Data was normalized relative to the housekeeping gene HGPRT. Analysis with one-way ANOVA revealed no significant differences between groups. (B) Western Blot analysis showing relative protein expression of PDE5, corrected for β-actin, of the three experimental groups in the dorsal hippocampus. Analysis with one-way ANOVA revealed no significant differences between groups. Bars represent the mean value (+ SEM) per group.
(TIF)

Figure S4 **Protein levels of synaptophysin and PSD95 in the dorsal hippocampus.** Western Blot analysis showing protein levels of synaptophysin (A) and PSD95 (B) of the three experimental conditions. Data was normalized relative to the housekeeping protein β-actin. Bars indicate mean (+ SEM) per group. One-way ANOVA revealed no statistical differences between the experimental groups.
(TIF)

Figure S5 **Protein levels of proBDNF and mBDNF in the dorsal hippocampus.** Western Blot analysis showing protein levels of proBDNF (A) and mBDNF (B) of the three experimental conditions. Data was normalized relative to the housekeeping protein β-actin. Bars indicate mean (+ SEM) per group. One-way ANOVA revealed no statistical differences between the experimental groups.
(TIF)

Figure S6 **Protein levels of pGSK-3β and β-catenin in the dorsal hippocampus.** Western Blot analysis showing protein levels of pGSK-3β (A), and β-catenin (B) of the three experimental groups. Data was normalized relative to the housekeeping protein β-actin. Bars indicate mean (+ SEM) per group. One-way ANOVA revealed no statistical differences between the experimental groups.
(TIF)

Figure S7 **Protein levels of ERK2 and pCREB in the dorsal hippocampus.** Western Blot analysis showing protein levels of ERK2 (A) and pCREB (B) of the three experimental conditions. Data was normalized relative to the housekeeping protein GAPDH. Bars indicate mean (+ SEM) per group. Significant differences between housing conditions are indicated with asterisks (Tukey HSD; *: p<0.05; **: p<0.01; ***: p<0.001).
(TIF)
