# Supplementary figures and images for: PDE5 Inhibition Improves Object Memory in Standard Housed Rats but Not in Rats Housed in an Enriched Environment: Implications for Memory Models?
Source: PLoS One. 2014 Nov 5;9(11):e111692. doi: 10.1371/journal.pone.0111692 (PMC4221101; doi:10.1371/journal.pone.0111692)

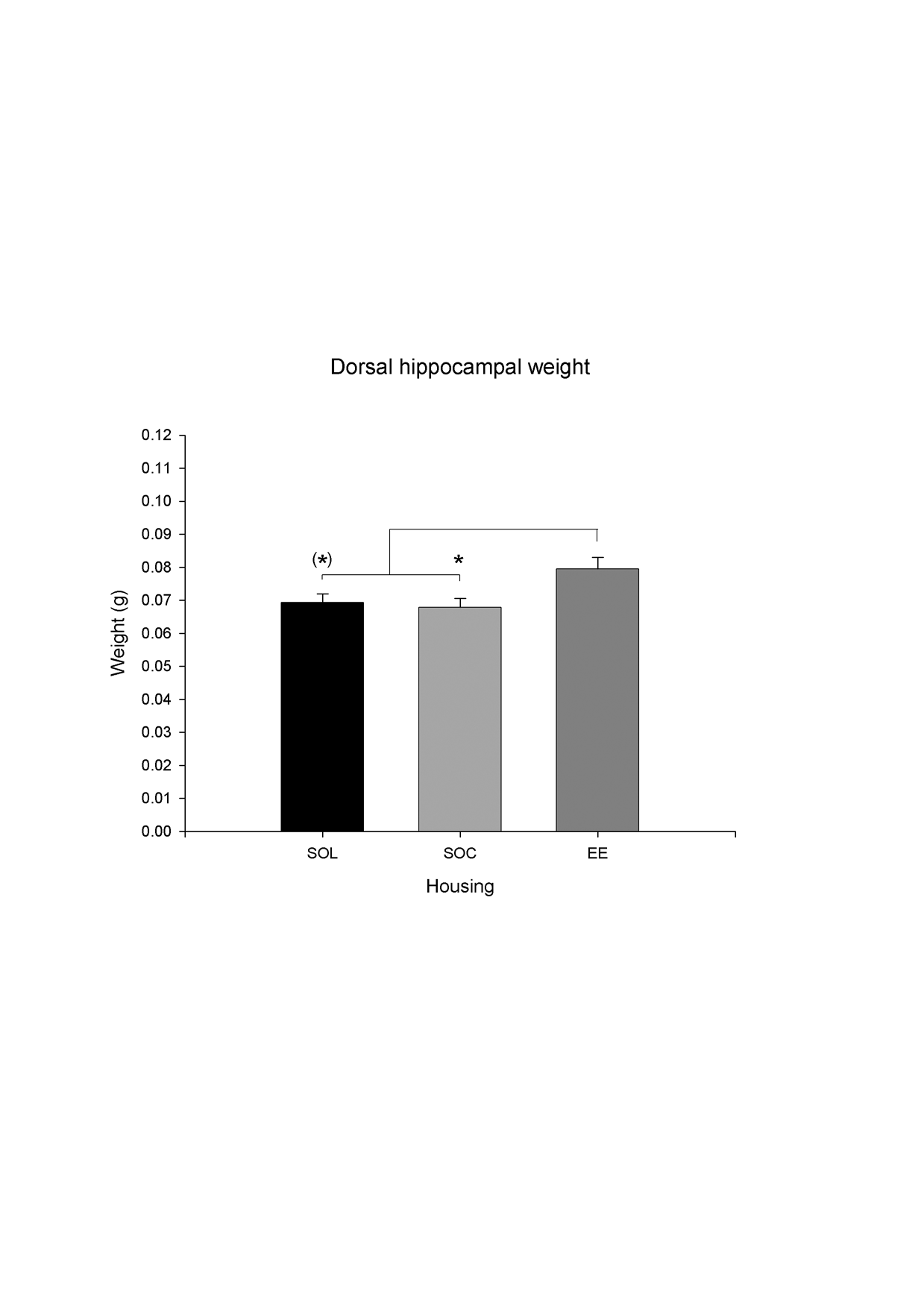

Supplement: Figure S1 — Dorsal hippocampal weights. Dorsal hippocampal weights of the three experimental groups in grams (mean + SEM). Significant differences between groups are indicated with asterisks (Tukey HSD; (*): p = 0.052; *: p<0.05). (TIF) [file pone.0111692.s001.tif]

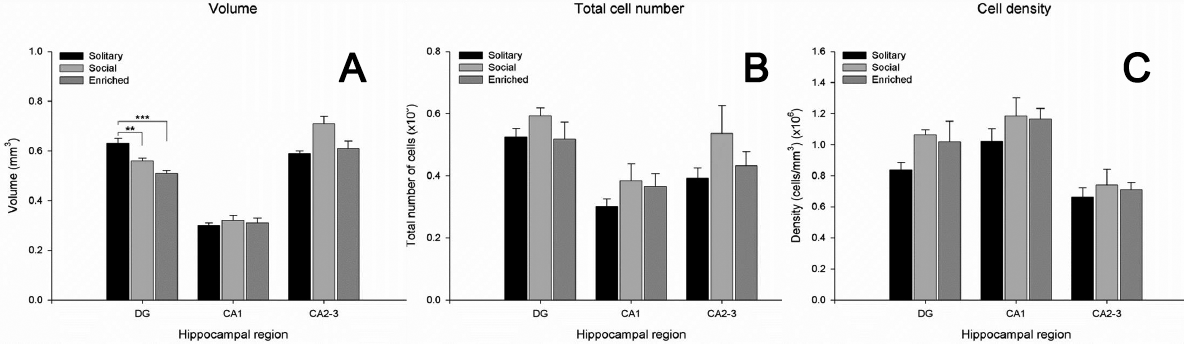

Supplement: Figure S2 — Stereology. The volume (mm3) total number of cells and cell density (number of cells/mm3) in the DG, CA1 and CA2–3 sub-regions of dorsal hippocampus of solitary- (SOL), social- (SOC) and environmentally enriched (EE) animals. One-way ANOVA only revealed significant differences between the housing conditions in the DG, these are indicated with asterisks (Tukey HSD; **: p<0.01; ***: p<0.001). (TIF) [file pone.0111692.s002.tif]

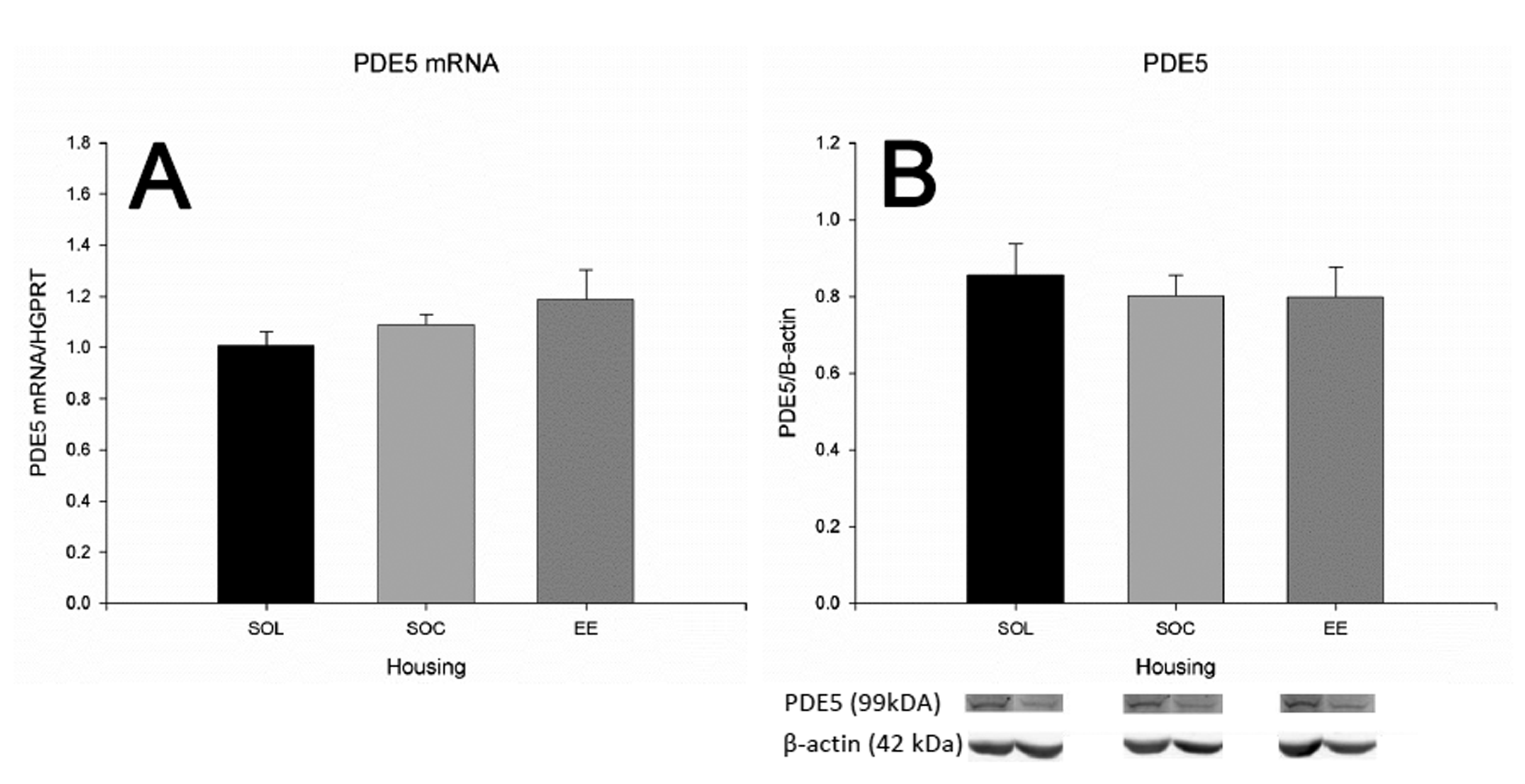

Supplement: Figure S3 — PDE5 mRNA and protein concentration in the dorsal hippocampus. (A) qPCR analysis showing mRNA levels of PDE5 of the three experimental conditions. Data was normalized relative to the housekeeping gene HGPRT. Analysis with one-way ANOVA revealed no significant differences between groups. (B) Western Blot analysis showing relative protein expression of PDE5, corrected for β-actin, of the three experimental groups in the dorsal hippocampus. Analysis with one-way ANOVA revealed no significant differences between groups. Bars represent the mean value (+ SEM) per group. (TIF) [file pone.0111692.s003.tif]

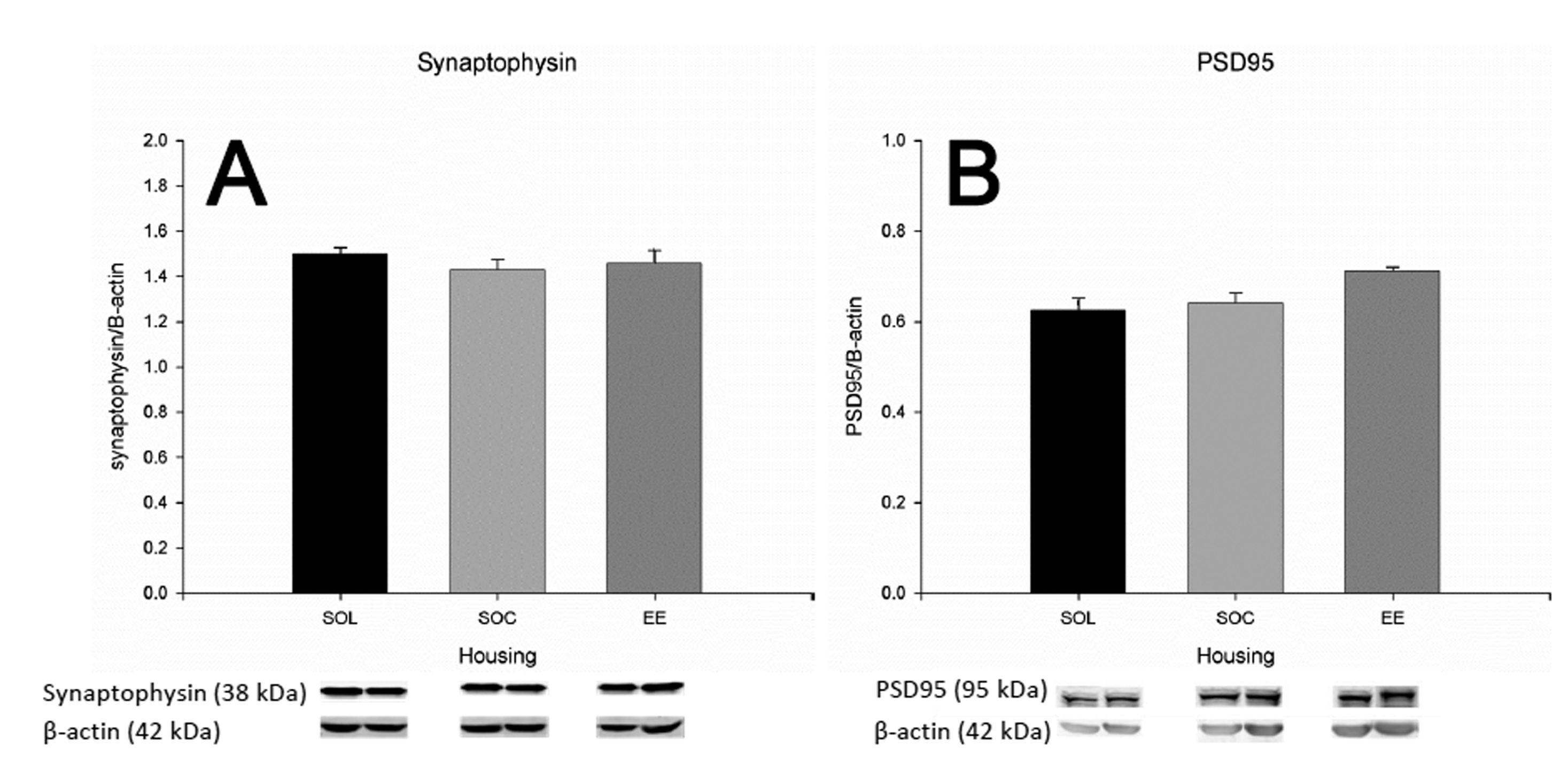

Supplement: Figure S4 — Protein levels of synaptophysin and PSD95 in the dorsal hippocampus. Western Blot analysis showing protein levels of synaptophysin (A) and PSD95 (B) of the three experimental conditions. Data was normalized relative to the housekeeping protein β-actin. Bars indicate mean (+ SEM) per group. One-way ANOVA revealed no statistical differences between the experimental groups. (TIF) [file pone.0111692.s004.tif]

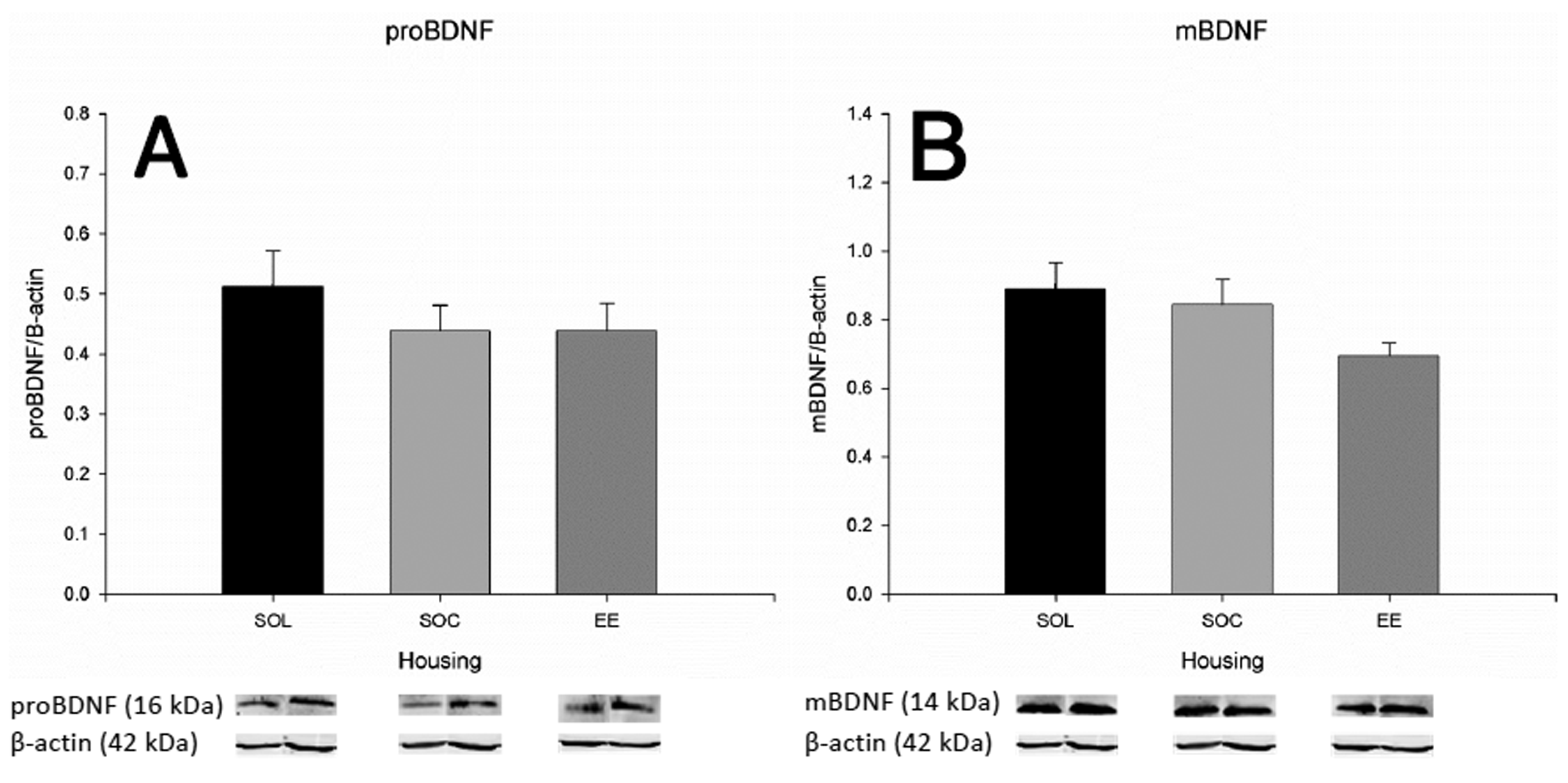

Supplement: Figure S5 — Protein levels of proBDNF and mBDNF in the dorsal hippocampus. Western Blot analysis showing protein levels of proBDNF (A) and mBDNF (B) of the three experimental conditions. Data was normalized relative to the housekeeping protein β-actin. Bars indicate mean (+ SEM) per group. One-way ANOVA revealed no statistical differences between the experimental groups. (TIF) [file pone.0111692.s005.tif]

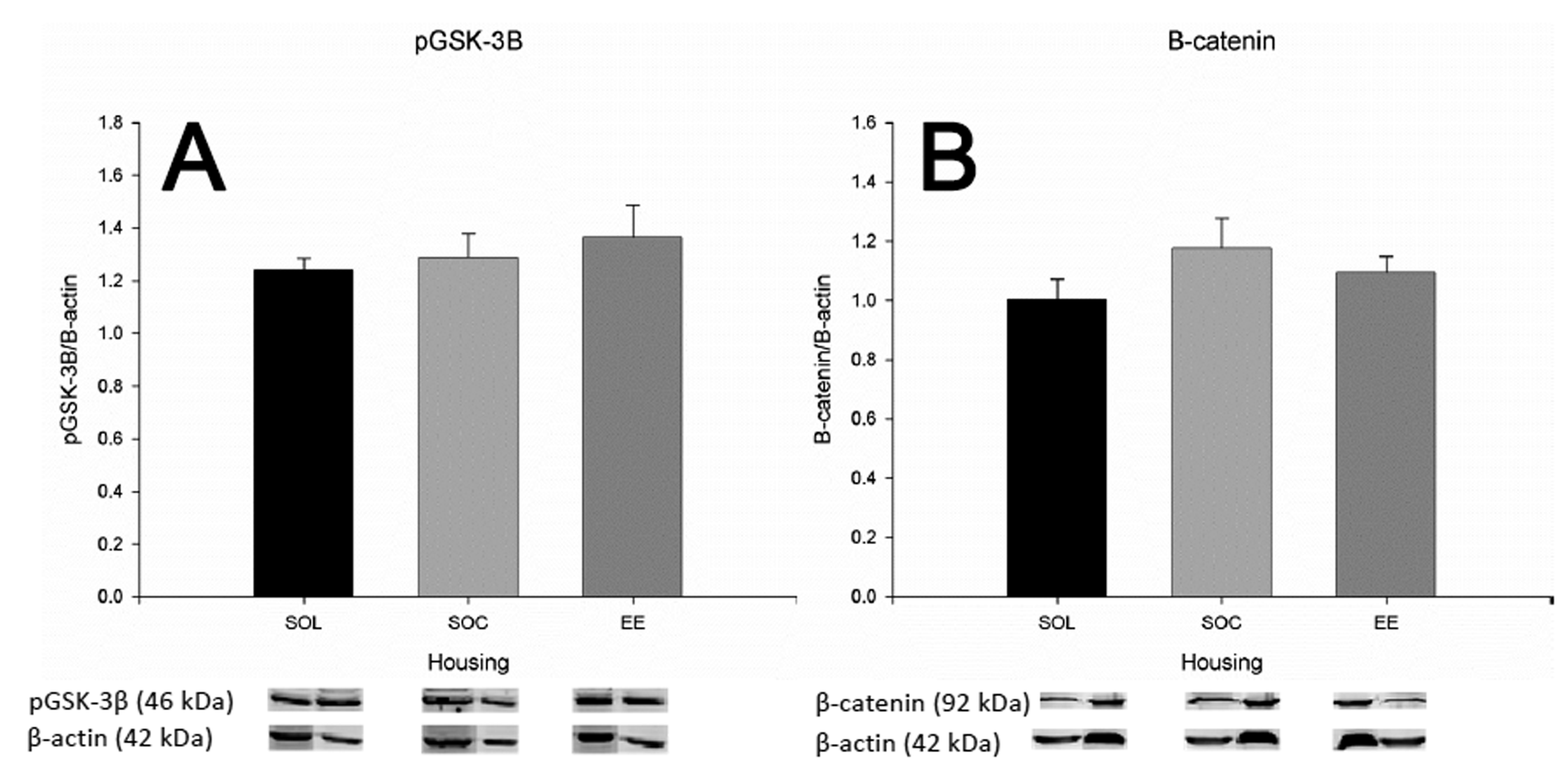

Supplement: Figure S6 — Protein levels of pGSK-3β and β-catenin in the dorsal hippocampus. Western Blot analysis showing protein levels of pGSK-3β (A), and β-catenin (B) of the three experimental groups. Data was normalized relative to the housekeeping protein β-actin. Bars indicate mean (+ SEM) per group. One-way ANOVA revealed no statistical differences between the experimental groups. (TIF) [file pone.0111692.s006.tif]

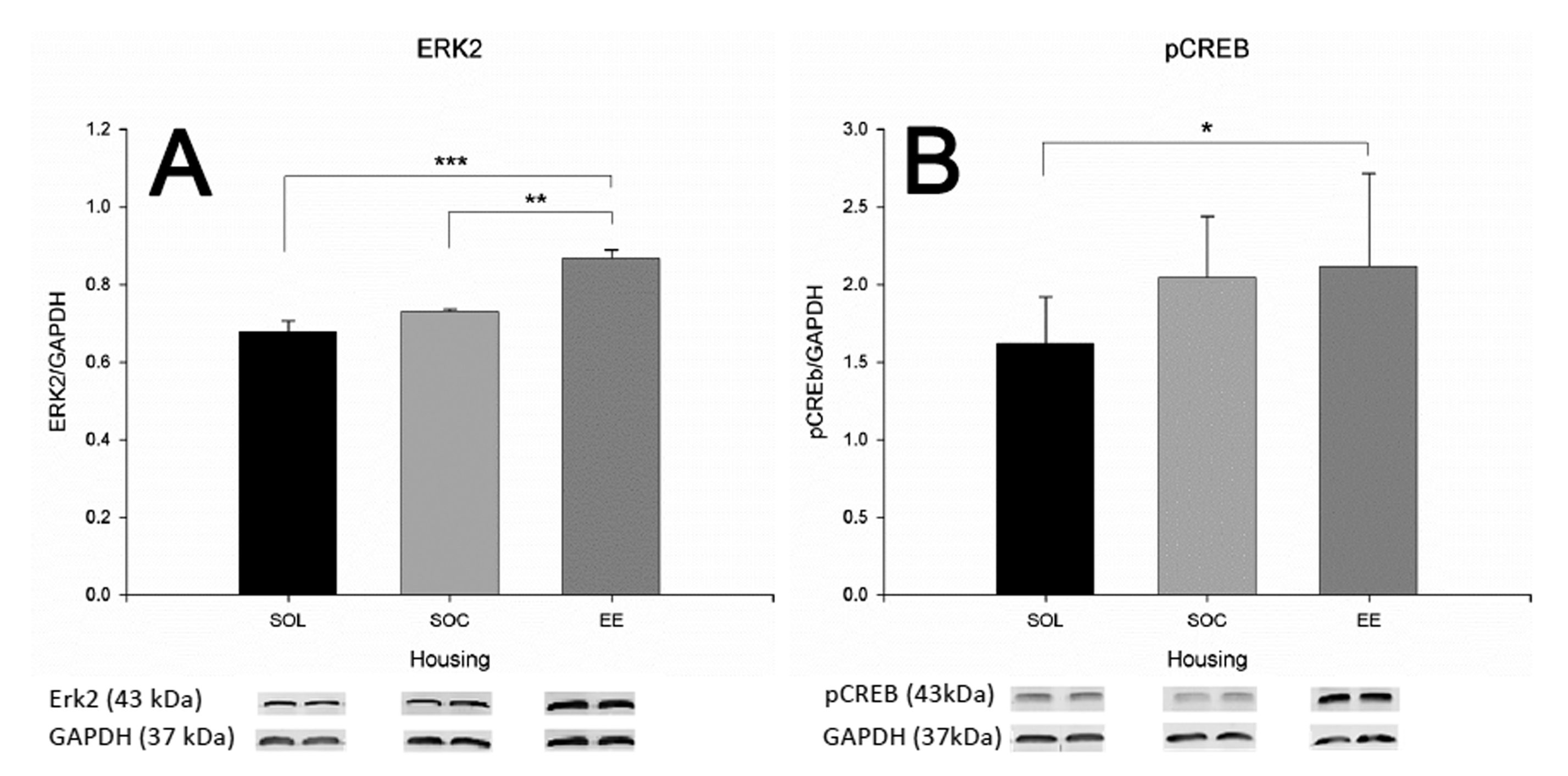

Supplement: Figure S7 — Protein levels of ERK2 and pCREB in the dorsal hippocampus. Western Blot analysis showing protein levels of ERK2 (A) and pCREB (B) of the three experimental conditions. Data was normalized relative to the housekeeping protein GAPDH. Bars indicate mean (+ SEM) per group. Significant differences between housing conditions are indicated with asterisks (Tukey HSD; *: p<0.05; **: p<0.01; ***: p<0.001). (TIF) [file pone.0111692.s007.tif]
